# Mechanisms of TREM2 mediated immunosuppression and regulation of cancer progression

**DOI:** 10.3389/fonc.2024.1375729

**Published:** 2024-04-25

**Authors:** Xia Lei, Ya Ni Gou, Jin Yong Hao, Xiao Jun Huang

**Affiliations:** Department of Gastroenterology, Second Hospital of Lanzhou University, Lanzhou, China

**Keywords:** immune, signal pathway, cancer, mechanism, therapy

## Abstract

Cancer immunotherapy has recently emerged as a key strategy for cancer treatment. TREM2, a key target for regulating the tumor immune microenvironment, is important in cancer treatment and progression. TREM2 is an immune signaling hub that regulates multiple pathological pathways. It not only suppresses anti-tumor immune responses by inhibiting T cell-mediated immune responses, but it also influences tumorigenesis by affecting NK cell-mediated anti-tumor immunity. Noticeably, TREM2 expression levels also vary significantly among different tumor cells, and it can regulate tumor progression by modulating various signaling pathways. Above all, by summarizing the role of TREM2 in cancer immunotherapy and the mechanism by which TREM2 regulates tumor progression, this paper clarifies TREM2’s role in both tumor progression and cancer therapy, identifying a new therapeutic target for oncology diseases.

## 1 Introduction

Triggering receptor expressed on myeloid cells 2 (TREM2) is a transmembrane immunoglobulin superfamily receptor that is primarily expressed in brain microglia and peripheral macrophages (1, 2) and serves as a central immune signaling hub for a variety of pathological pathways. TREM2 lacks signaling capacity and instead propagates signals intracellularly by interacting with ligands, binding to, and phosphorylating the adaptor proteins DNAX activation protein (DAP) 12 and DAP 10. DAP12, also known as tyrosine kinase binding protein (TYROBP), and DAP10 can activate spleen tyrosine kinase (Syk) and phosphatidylinositol 3-kinase (PI3K), respectively (2), which mediate downstream signaling and are involved in immunoinflammatory responses in the pathophysiology of various diseases. Previously, TREM2 was extensively studied and identified as a surface receptor required for microglia response to neurodegenerative changes, which has been associated with the pathogenesis of Alzheimer’s disease (AD) and other neurodegenerative diseases (NDDs) (3). Mutations or deletions of the TREM2 gene reduce microglia’s ability to clear AD-related β-amyloid and tau proteins, increase the diffusion of neuroinflammatory dystrophies and tau proteins around plaques, and shift microglia from a homeostatic to a pathological state that promotes the onset of AD (4). Furthermore, TREM2 is also linked to the development of metabolic diseases, and TREM2 deficiency inhibits the downstream pathway of lipid-associated macrophages (LAMs) (5), which plays an important role in the onset and progression of hypercholesterolemia, atherosclerosis, and nonalcoholic fatty liver disease (6–8).

In recent years, TREM2 has been discovered to be widely expressed on the surface of monocyte-macrophage lineage cells as well, and it is a marker for tumor-associated macrophages (TAMs) in a variety of tumor types (9). Besides, TREM2 is widely expressed on tumor cells and can regulate tumor cell proliferation and metastasis through various signaling pathways, thereby influencing tumor progression (10). Moreover, TREM2 plays an immunosuppressive role in tumor microenvironment (TME), which can negatively regulate anti-tumor immune response and assist tumor cell immune escape (11). Blocking the specific ligands of TREM2 may be a major advancement in cancer immunotherapy and is expected to be a new target for tumor immunotherapy.

In conclusion, in this review, we evaluate the research progress of TREM2 in immunotherapy, with a focus on its effects on T cells, macrophages and NK cells. In addition to this, we also specifically highlight the expression levels of TREM2 in different tumor tissues, as well as the pathways by which TREM2 promotes or inhibits cancer progression. These findings provide a strong theoretical foundation for TREM2’s emergence as a new target for tumor immunotherapy, and targeting TREM2 could be a new way to treat tumor diseases.

## 2 TREM2 and immunotherapy

### 2.1 TREM2 and T cell

**Figure 1:**
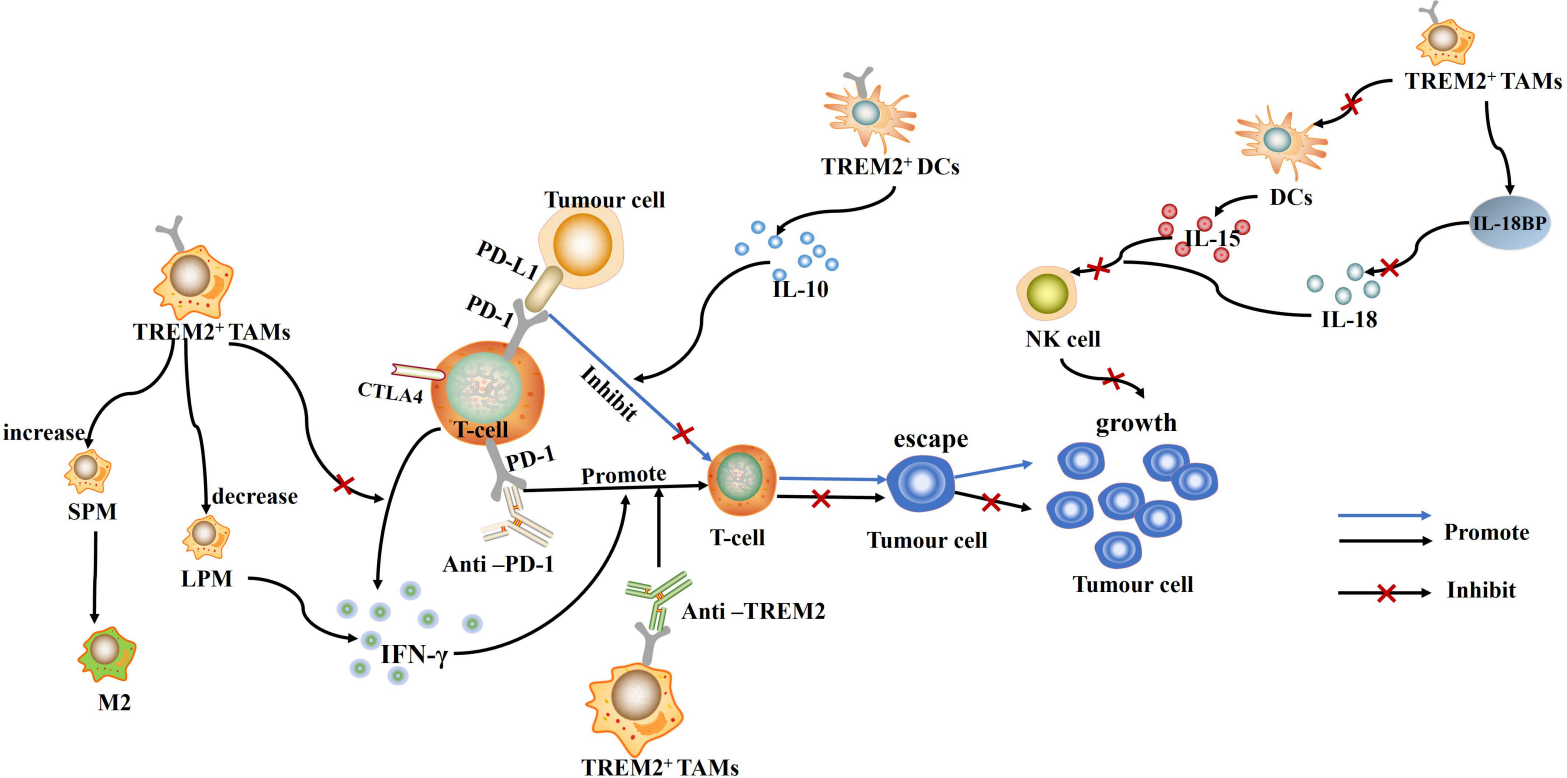
TREM2^+^ TAMs and TREM2^+^ DCs can inhibit T cell function by direct or indirect means, which in turn promotes immune escape and tumor growth. Conversely, Anti-PD-1 and Anti-TREM2 can promote T cell-mediated immune response, inhibit immune escape of tumor cells, and suppress tumor growth.

T cell-mediated immune response plays a crucial role in anti-tumor immunity (12). Immune checkpoints such as cytotoxic T- lymphocyte associated antigen-4 (CTLA-4) and programmed death-1 (PD-1) can induce an immunosuppressive microenvironment by negatively regulating T-cell-mediated immune responses (11). Among them, the PD-L1/PD-1 axis plays a key role in tumor immune escape. Programmed death ligand 1 (PD-L1) is highly expressed on the surface of malignant tumor cells. It can inhibit T cell response by binding to PD-1 expressed on the surface of T cells, leading to tumor evasion of T cell immunity (13). With the use of immune checkpoint inhibitors (ICIs), PD-1 inhibitors (PD-1/PD-L1 inhibitors), blocks the interaction between PD-L1 and PD-1, removes the inhibitory effect on T-cells, and controls tumor growth (14). Yet, ICIs are not entirely effective in antitumor therapy (**Figure 1**).

In recent years, TREM2, a myeloid receptor, has been found to play an important role in altering tumor myeloid infiltrates as well as decreasing the efficacy of immunotherapy (15). In a variety of cancers, infiltration of TREM2^+^ myeloid cells is associated with the formation of an immunosuppressive microenvironment (16). TREM2^+^ TAMs can inhibit the proliferation of CD8^+^ T cells by expressing CD107a, perforin 1, and tumor necrosis factor alpha (TNF-α), and suppress the effector function of CD8^+^ T cells (17). In addition, TREM2^+^ LAMs can also interact with Treg cells via the CCL20/CXCL9/CXCL10/CXCL12-CXCR3 axis, recruiting suppressor Treg cells, inhibiting the function of effector T cells, and promoting tumor microenvironment remodeling (18). These findings provide a mechanistic link for TREM2 to promote the formation of an immunosuppressive microenvironment. Blocking or targeting TREM2 gene can change the tumor immune environment by enhancing T-cell effector function, enhancing the effect of anti-PD-1 immunotherapy (19).

In uroepithelial carcinoma (UC), the abundance of TREM2^+^ macrophages is significantly higher and associated with a lower overall survival rate (20). And, TREM2^+^ macrophages inhibit T cell function and reduce the anti-tumor capacity of CD8^+^ T cells, which leads to poor response of UC to ICIs treatment (20). Similarly, TREM2 is highly expressed in glioblastoma (GBM), and its expression is consistent with the expression of inhibitory immune checkpoints such as PD-L1, PD-L2, and Galectin-9, suggesting that TREM2 may inhibit T cell-mediated immune responses by regulating the expression of immune checkpoints, thereby promoting glioma cell immune escape (21). Inhibiting TREM2 function can promote T cell infiltration and activation, increase PD-1^+^ CD8^+^ T cells in the TME, and thus improve the efficacy of anti-PD-I therapy (22).

Tumor-induced dendritic cells (DCs) can also directly inhibit T-cell proliferation by secreting immunosuppressive cytokines such as IL-10 or TGF-β (23). In non-small cell lung cancer (NSCLC) cells, TREM2^+^ DCs are heavily infiltrated in cancerous tissues and can inhibit T cell proliferation by secreting large amounts of IL-10 (24), promoting tumor growth (**Figure 1**). Mechanistically, TREM2 can recruit Syk and initiate signaling cascades by binding to DAP12 (25), leading to IL-10 secretion. The use of Syk inhibitors can inhibit IL-10 production by TREM2^+^ DCs suggesting that TREM2 acts as a negative immunomodulator through the Syk pathway in an IL-10-dependent manner. All in all, TREM2 can promote tumor growth by promoting T-cell dysfunction and tumor immune escape, and is an important pathology-inducing immune signaling hub (26).

Although ICIs are an effective immunotherapy, identifying the underlying causes of ICI resistance remains a challenge. According to studies, overexpression of TREM2 in macrophages may be associated with ICIs resistance. In ICIs non-responders, macrophages overexpressing TREM2 show a unique gene expression pattern and overexpress key genes of the complement system (C3, C1QA, C1QB, and C1QC) and M2 polarization genes, which block the anti-tumor activity of ICIs, leading to ICIs resistance (27). Cystatin C (CyC), a secreted cysteine protease inhibitor, is associated with the recruitment of TREM2^+^ macrophages. Increasing CyC can promote the migration or expansion of TREM2^+^ macrophages, thereby reducing the efficacy of ICIs. Targeting CyC may be an effective target to enhance the efficacy of cancer immunotherapy (28).

### 2.2 TREM2 and macrophage

Macrophage reprogramming is also one of the promising therapeutic strategies in current cancer treatment. Under the influence of TME cytokines, macrophages can also differentiate into different subtypes of TAMs, which are mainly classified into M1 and M2 types. Many studies have found that TREM2 is highly expressed on TAMs, and that M2 polarization genes such as MMP14, CD276 are also overexpressed in TREM2^+^ macrophages (27). This makes it possible that TREM2^+^ macrophages are functionally similar to M2 polarized macrophages, which may have pro-tumorigenic effects (27). Targeted inhibition of TREM2 can affect the phenotype and function of TAMs (29) and enhance the efficacy of tumor immunotherapy. In NSCLC, TREM2^+^ TAMs exhibit immunosuppressive effects. NSCLC patients with high TREM2^+^ TAMs infiltration exhibit late staging, poor prognosis, and unique NSCLC molecular features, especially EGFR mutations (17). Knockdown of TREM2 can reduce the polarization of M2 phenotype, causing TAMs to remodel into the M1 type, which exhibits a pro-inflammatory and immunostimulatory state, inhibits the growth as well as invasiveness of hepatic cell carcinoma (HCC) (30) and glioma cells (21), and suppresses tumor progression.

TAMs, as a major component of TME, can also play a tumorigenic role by promoting angiogenesis, secreting tumor growth factors, and promoting tumor invasion and metastasis (31). TREM2 is highly expressed on TAMs in a variety of cancers, including colorectal, triple-negative breast, and pancreatic cancers (15, 32–34), and is negatively correlated with survival, and is a pro-tumorigenic marker for TAMs in mouse models and human tumors. In bladder cancer (BLCA), TREM2 expression is also associated with tumor progression and decreased immunotherapy efficacy, and TREM2 may play a role by promoting epithelial mesenchymal transition (EMT) and T-cell exhaustion (35). In addition, TREM2^+^ TAMs are enriched and infiltrated in the invasive margin of early lung metastasis of breast cancer. They can inhibit anti-tumor immunity in metastatic foci and alter TME at the invasive margin by inhibiting T cell activation and IFN-γ secretion, thereby promoting tumor growth and progression (34).

Notably, TREM2, as a major immune checkpoint on TAMs (36), has also been linked to depletion of CD8^+^ tumor-infiltrating lymphocytes (TILs) and anti-PD-1 resistance in human cancers (17, 32, 37). This is true for patients with esophageal squamous cell carcinoma (ESCC) (38) and melanoma (39). Higher TREM2^+^ TAMs infiltration is associated with poor overall survival and insensitivity to anti-PD-1 monoclonal antibody immunotherapy. By using anti-TREM2 monoclonal antibody treatment, tumor growth can be inhibited and a strong anti-tumor immune effect can be produced, which enhances the activation function of CD8^+^ TIL and anti-PD-1 immunotherapeutic response (15). This suggests that TREM2 could be a very attractive target for immunotherapy modulation. Furthermore, in mesothelioma, TREM2 deficiency can lead to a decrease in monocyte-derived small peritoneal/pleural macrophages (SPM) and a compensatory increase in tissue-resident large peritoneal/pleural macrophages (LPM) (40). Considering that SPM preferentially promotes an M2-like phenotype, whereas LPM more specifically promotes the immune response and activates the IFN-γ response, making it more effective in activating the T cell response (40). Thus, the compensatory increase in LPM due to TREM2 deletion can explain in a side-by-side manner why TREM2 deletion is associated with enhanced CD8^+^ T-cell infiltration in tumors and more effective anti-PD-1 therapy (**Figure 1**).

### 2.3 TREM2 and NK cell

TREM2 can also modulate tumorigenesis by affecting NK cell-mediated anti-tumor immunity. Deletion of the TREM2 gene significantly reduces immunosuppression in TME in vivo, including a decrease in dysfunctional CD8^+^ T cells (expressing PD-1 and Tim-3) and an increase in NK cells and cytotoxic T cells (41). And the lack of cytotoxic NK cells in tumors is a feature of TME, which allows many cells to escape surveillance, and in various cancers, the reduction of NK cells can promote tumor growth (42–44). In non-small cell lung cancer (NSCLC), TREM2 expressed on monocyte-macrophages can not only block IL-18 production and signaling by enhancing interleukin (IL)-18/IL- 18bp decoy interactions, but also inhibit IL-15 production by DCs, which in turn inhibits NK cell recruitment and activation, suppresses NK cell-mediated anti-tumor immunity, and prevents NK cells from effectively killing tumor cells (36). Conversely, combining TREM2 blockers with exogenous NK cell enhancers will further enhance the anti-tumor response and enhance the therapeutic effect of anti-tumor immunity. In contrast to the above studies, TREM2 has been shown to play an important role in the differentiation and effector function of NK cells. Overexpression of TREM2 promotes the differentiation of NK cells and enhances their killing activity against tumor cells by activating the PI3K/Akt signaling pathway (45, 46). This difference in outcome may be due to the alteration of different signaling pathways by TREM2 and the exact mechanism remains to be further explored.

## 3 TREM2 and cancer

### 3.1 As a tumor promoter

**Figure 2:**
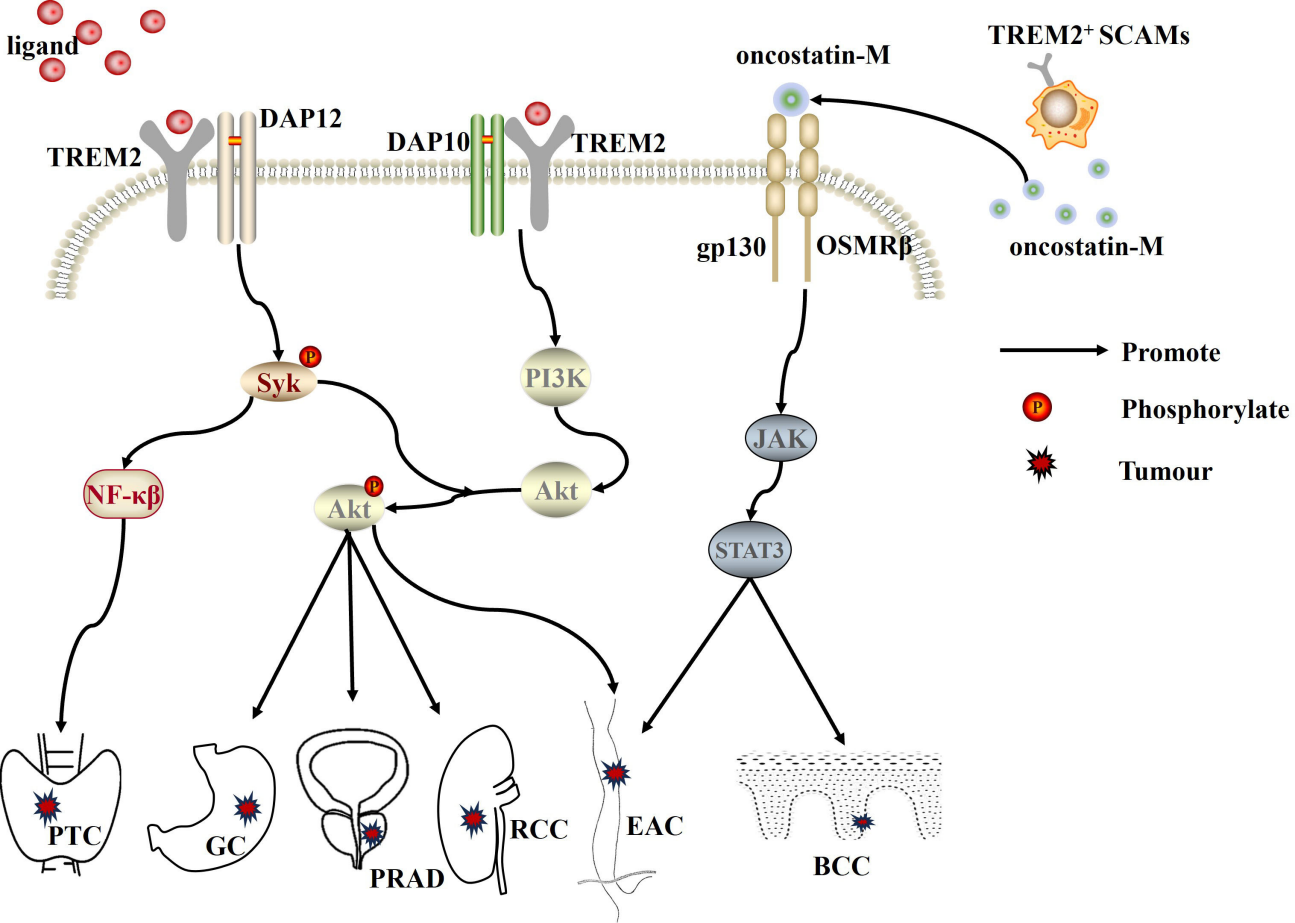
TREM2, as an oncogene, promotes the progression of PTC, GC, PRAD, RCC, EAC, and BCC by activating the PI3K/Akt, NF-κB, and JAK/STAT3 signaling pathways, respectively. PTC, papillary thyroid carcinoma; PRAD, prostate cancer; RCC, renal cell carcinoma; GC, gastric cancer; EAC, esophageal adenocarcinoma; BCC, basal cell carcinoma; SCAMs, skin cancer-associated macrophages.

The expression level of TREM2 is significantly upregulated in tumor tissues of patients with gastric cancer (GC) (47), oral squamous cell carcinoma (OSCC) (48), lung cancer (49), papillary thyroid carcinoma (PTC) (50), renal cell carcinoma (RCC) (51), and prostate cancer (PRAD) (52). Moreover, high levels of TREM2 are significantly correlated with the extent of tumor invasion, tumor-node-metastasis (TNM) stage, and histological grading. Highly expressed TREM2 promotes the proliferation, migration, and invasion of GC (53), PRAD (52) and RCC (51) through activation of the PI3K/Akt signaling pathway. It also activates the NF-κB pathway, promoting the onset and development of PTC (50). In addition to this, in basal cell carcinoma (BCC) of the skin, a large number of TREM2^+^ skin cancer-associated macrophages (SCAMs) are enriched and infiltrated (54). SCAMs can also promote tumor epithelial cell proliferation and tumor growth in an immunosuppressive non-dependent manner by secreting the ligand oncostatin-M (OSM), which induces JAK/STAT3 signaling. Similarly, in esophageal adenocarcinoma (EAC), TREM2 overexpression can promote the growth of EAC cells by activating DAP12/Syk/AKT or JAK/STAT3 signaling pathways (55). These findings suggest that TREM2 can promote tumorigenesis and progression by activating different signaling pathways, indicating that it is an important oncogene (**Figure 2**).

### 3.2 As a tumor suppressor

**Figure 3:**
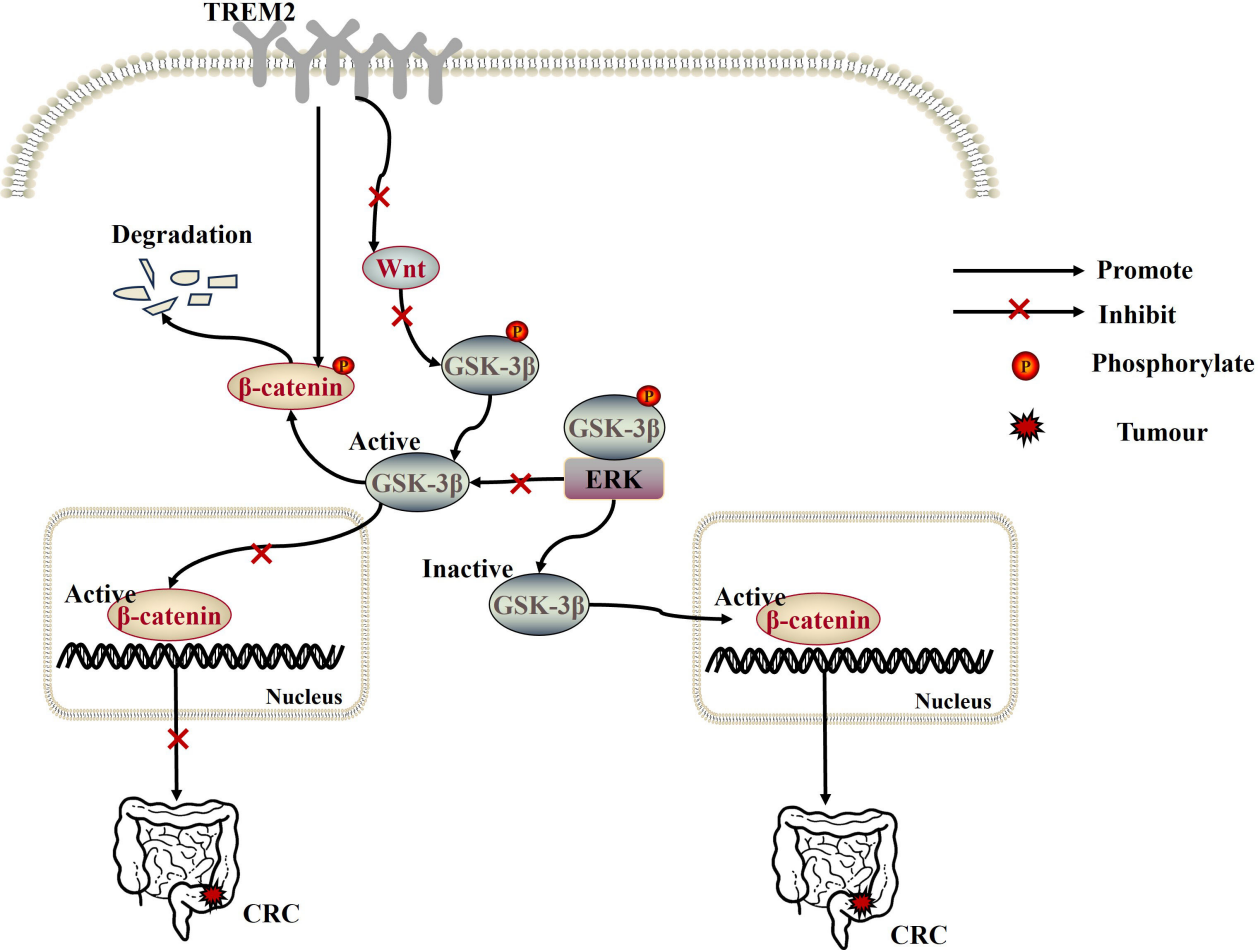
Overexpression of TREM2 not only inhibits CRC progression by negatively regulating the Wnt/ERK/GSK-3β signaling pathway, but also acts as a β-catenin regulator to inhibit CRC tumor progression by promoting the proteasomal degradation of β-catenin in the cytoplasm and negatively regulating the Wnt/β-catenin signaling pathway.

However, in colorectal cancer (CRC) tissues, TREM2 expression decreases progressively with increasing tumor stage and plays a tumor-suppressive role in CRC. Overexpression of TREM2 inhibits CRC progression by negatively regulating the Wnt/ERK/GSK-3β signaling pathway (**Figure 3**), as well as inhibiting CRC cell proliferation, invasion, and metastasis by down-regulating the expression of cyclin D1 and MMP9 (matrix metalloproteinase 9) (56). Notably, TREM2 can also act as a β-catenin regulator by promoting the proteasomal degradation of β-catenin in the cytoplasm (57) and negatively regulating the Wnt/β-catenin signaling pathway, which in turn inhibits CRC tumor progression in vivo and in vitro (58). Similarly, the expression level of TREM2 is upregulated in skin cutaneous melanoma tissues. And, high expression of TREM2 is associated with tumor-infiltrating immune cells (e.g., macrophages, B cells, CD8^+^ T cells, CD4^+^ T cells, DCs, etc.) and longer cumulative survival, playing a protective role in skin cutaneous melanoma (39). Besides, in acute myeloid leukemia (AML), TREM2, as a novel receptor for IL-34, can induce myeloid differentiation and inhibit AML progression by inhibiting the ERK1/2/Rasal3 signaling pathway. On the contrary, TREM2 gene-deficient AML cells and normal myeloid cells exhibit resistance to IL-34 treatment and are associated with poor prognosis (59). These findings suggest that TREM2 can inhibit tumorigenesis and development by activating different signaling pathways and plays an important tumor suppressor role.

### 3.3 Dual role

#### 3.3.1 HCC

**Figure 4:**
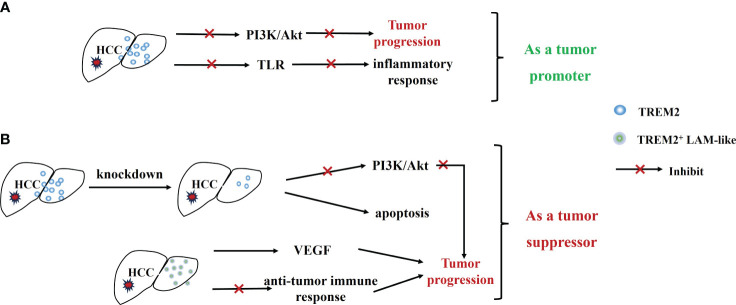
Dual role of TREM2 in HCC. **(A)** Highly expressed TREM2 inhibits tumor progression and inflammatory response through inhibition of the PI3K/Akt signaling pathway and TLR. **(B)** TREM2^+^ LAM-like promotes cancer progression by promoting tumor angiogenesis and suppressing anti-tumor immune responses, and knockdown of TREM2 inhibits Tumor progression.

TREM2 is predominantly expressed on non-substantial hepatocytes in liver tissues, and it is significantly upregulated in HCC tissues (60). However, TREM2 expression is significantly reduced with tumor progression, especially in metastatic HCC, and such reduction is associated with a poorer prognosis as well as aggressive pathological features (61). Furthermore, TREM2 can also play a protective role in HCC by inhibiting toll-like receptor 4 (TLR4)-induced inflammatory responses (62, 63), reducing activated proteins downstream of TLR4 (such as p38-MAPK and ERK) to reduce inflammation levels and suppress chronic inflammation (60). Similar to the above findings, in a study by Tang W et al (61), TREM2 also acted as a tumor suppressor. Overexpression of TREM2 can inhibit the occurrence of EMT by targeting the PI3K/Akt/β-Catenin pathway, thereby inhibiting the invasion and metastasis of tumor cells (**Figure 4A**).

However, some studies have presented a different viewpoint, suggesting that TREM2 is highly expressed in cancerous tissues (64) and plays a promotional role in the development and progression of HCC. Overexpression of TREM2 promotes tumorigenesis by activating the PI3K/AKT signaling pathway (65). Knockdown of TREM2 gene can inhibit the proliferation of HCC cells by targeting caspase/Bcl and CDK1 signaling pathways (64). Besides, TREM2 is also highly expressed on LAMs (18) and is enriched and infiltrated in HCC. TREM2^+^ LAMs not only promote tumor angiogenesis by facilitating VEGF signaling, but also promote cancer progression by suppressing anti-tumor immune responses, correlating with poor clinical outcomes in HCC patients (18) (**Figure 4B**).

In conjunction with the above research, we hypothesized that these two different roles of TREM2 may be complementary and coordinated in HCC tissues. They limit inflammatory damage and tumorigenesis in the early stages, but suppress anti-tumor immune responses and promote cancer progression later on. In conclusion, the mechanism of TREM2’s role in HCC still needs to be further explored.

#### 3.3.2 Glioma

TREM2 also plays a dual role in gliomas. Gliomas are the most common adult primary brain tumors, accounting for more than 80% of central nervous system (CNS) malignancies. Among them, glioblastoma (GBM) is the highest-grade glioma with the worst clinical prognosis. Several studies have shown that the expression levels of TREM2 in glioma tissues are significantly increased, and it is an oncogene. Increased expression of TREM2 can promote cell proliferation, migration, and invasion, and it is strongly related to pathological grade and negatively related to overall survival of glioma patients (66). Remarkably, GBM tumors are also infiltrated with a large number of TREM2^+^ myeloid cells (56). Inhibiting the TREM2 gene can trigger the anti-tumor activity of GBM myeloid cells, increase interferon-γ induced immune activation, and promote pro-inflammatory phenotypes, ultimately inhibiting tumor growth and prolonging survival (22). In addition, TREM2 was found to be highly expressed in glioma-associated microglia/macrophages (GAMs) in glioma tissues and had a negative correlation with patient survival time (67). However, interestingly, TREM2 gene deficiency can, on one hand, inhibit genes in MHC I and II clusters (MHC I cluster: H2-D1, H2-M3, H2-Q4, etc.; MHC II cluster: H2-Ab1, H2-DMb1, H2-Eb1), inhibit antigen presentation, which in turn may lead to immune escape and impaired function of CD4^+^/CD8^+^ cells and NK cells, and on the other hand, TREM2 gene deficiency can also inhibit the expression of immune genes with pro-angiogenic functions, such as Ccl5, Ccr5, Ccl12, Icam1, and Itgal, in glioma tissues, thereby inhibiting angiogenesis in glioma tissues and suppressing tumor growth and progression (67).

Besides, it has also been shown that, unlike in other cancers, TREM2 expression is not associated with immunosuppressive pathways in glioma TME, but rather with phagocytosis and is an important immunomodulator (68). High levels of TREM2^+^ monocytes can reduce tumor load by enhancing tumor phagocytosis and inhibiting inflammatory responses, and exhibit longer survival (69, 70). It is suggested that TREM2^+^ monocytes play a protective role in gliomas. Therefore, enhancing the potential of myeloid cells in TME to phagocytose tumor cells by inducing TREM2-mediated phagocytosis could be a potential immunotherapeutic strategy for the treatment of brain tumors.

## 4 Conclusion and outlook

Nowadays, as people’s lifestyles change, the incidence of cancer rises, making it difficult to find an effective target for cancer therapy. TREM2, as an immune signaling hub mediating multiple pathological pathways, is widely expressed on immune cells with important pro-immunosuppressive effects, and plays a great role in cancer immunotherapy. In TME, the expression of TREM2 on TAMs as well as DCs can promote tumor growth by influencing the T cell-mediated immune response and facilitating immune escape from tumors. Targeted treatment with anti-TREM2 monoclonal antibody can produce potent anti-tumor immune effects, inhibit tumor growth, and also enhance the anti-PD-1 immunotherapeutic response and improve the therapeutic effect of ICIs, making it an effective target for cancer immunotherapy.

Furthermore, the expression level of TREM2 in tumor cells is also closely related to tumor progression. In different tumor cells, TREM2 expression levels vary significantly and can promote or inhibit tumor progression through different signaling pathways, such as NF-κB, JAK/STAT3 and PI3K/Akt signaling pathways. Among them, the PI3K/Akt signaling pathway is an important signaling pathway for TREM2 to regulate tumorigenesis. Highly expressed TREM2 can activate or inhibit the PI3K/Akt signaling pathway to regulate the occurrence and development of tumors. In GC, PRAD, and RCC tissues, TREM2 can promote tumor cell progression by activating the PI3K/Akt signaling pathway. In contrast, in CRC tissues, TREM2 inhibits the proliferation and metastasis of CRC tumor cells by inhibiting the PI3K/Akt signaling pathway. It is noteworthy that, in HCC tissues, TREM2 can both activate the PI3K/Akt signaling pathway to promote tumor progression and inhibit tumor progression by inhibiting the PI3K/Akt/β-Catenin pathway. These two different effects exhibited by TREM2 on the PI3K/Akt signaling pathway may be related to different cell types and cell states. Therefore, more studies are needed in the future to verify the mechanism of TREM2’s regulatory effects on different tumor cells.

To summarize, TREM2 plays an important role in tumor progression and cancer immunotherapy, and targeting TREM2 may be an appealing target for immunotherapy. Because of the tissue variability of TREM2 expression, more studies are needed in the future to explore the expression of TREM2 in different cancers as a way to better improve the efficacy of cancer therapy and control tumor progression.

## Author contributions

XL: Conceptualization, Writing – original draft, Writing – review & editing. YG: Conceptualization, Writing – original draft, Writing – review & editing. JH: Writing – review & editing. XH: Supervision, Writing – review & editing.
